# Stress in family caregivers and professional care workers providing home care to individuals with ADRD

**DOI:** 10.1002/alz70858_104510

**Published:** 2025-12-25

**Authors:** Eunbee Liu, Azita Emami, Gabriella Engstrom, Olimpia Paun, Jaehyuk Song, Hyejin Kim

**Affiliations:** ^1^ Rush University College of Nursing, Chicago, IL, USA; ^2^ Yale University School of Nursing, Orange, CT, USA; ^3^ Karonliska Institutet Department of Neurobiology, Care Sciences and Society, Huddinge, Stockholm, Sweden; ^4^ Florida Atlantic University Charles E. Schmidt College of Medicine, Boca Raton, FL, USA; ^5^ Rush University Medical College, Chicago, IL, USA

## Abstract

**Background:**

In‐home caregiving for individuals with Alzheimer's disease and related dementias (ADRD) significantly increases caregiver stress. While both family members and professional caregivers face substantial challenges, there is limited understanding of how they perceive their stress. This secondary analysis examined the differences and similarities in perceived stress among family caregivers and home care workers, both frontline caregivers who may experience varying levels of stress while providing care at home.

**Method:**

We analyzed baseline data from 41 family caregivers and 17 home care workers, all primary caregivers for individuals with ADRD. Data for family caregivers were collected from a study in Sweden that focused on a music‐based intervention for individuals with ADRD and their co‐residing family caregivers. Home care worker data came from a study on wearable technology among Korean immigrants. Stress was measured using the 10‐item Perceived Stress Scale (PSS‐10) in its Swedish and Korean versions, respectively. Responses were rated on a 5‐point Likert scale, with coping subscale items reversed to reflect higher stress. Independent samples t‐tests were performed to compare total PSS‐10 scores (range: 0‐40), stress subscale scores (range: 0‐24), and coping subscale scores (range: 0‐16) between family caregivers and home care workers.

**Result:**

Family caregivers (mean age: 74.88, range: 37‐90) were significantly older than home care workers (mean age: 61.94, range: 52‐75) (*p* < .01). While all family caregivers managed care for one individual, five home care workers (29.41%) cared for more than one individual. Family caregivers reported significantly higher stress levels (M±SD=15.66±7.02) compared to home care workers (M±SD=11.94±3.88) (*p* = .045). Specifically, family caregivers had significantly higher stress subscale scores (M±SD=10.56±5.54) than home care workers (M±SD: 7.35±3.06) (*p* = .029). However, no significant differences were observed in coping subscale scores (*p* > .05).

**Conclusion:**

The findings revealed that family caregivers experienced higher stress levels with older age, compared to home care workers. This suggests the need for tailored interventions to address the elevated stress and improve their well‐being.

